# The origin and function of arbitrary signals: Making false statements, having long hair, and smoking Virginia Slims

**DOI:** 10.1093/pnasnexus/pgae408

**Published:** 2024-09-14

**Authors:** Birger Wernerfelt

**Affiliations:** J. C. Penney Professor Emeritus, MIT Sloan School of Management, MIT, Cambridge, MA 02142, USA

**Keywords:** social signals, fake news, personal brand

## Abstract

We propose a model in which players take actions that run counter to social norms in part to announce their stand on a social controversy but also, and maybe mostly, to gain image benefits that allow them to join groups that are socially attractive to them. We give several examples, but the “election denial” debate is an important application: rather than assuming that proponents believe their claims to be true, it suggests that false statements can serve as symbolic actions and help them engage in self-branding for social and psychological gain. Specifically, the willingness to make a controversial statement can be a credible signal because untruth is ill-received by some members of society and therefore entails some costs. It is immaterial whether election deniers believe their claim to be true, but it is important that some members of society believe that it is false and therefore think poorly of those who make it: if there is social consensus about the truth of a statement, it cannot serve a signaling function. The same mechanism explains several other verbal and nonverbal signals associated with different sides of social controversies and analysis of those helps clarify the mechanism.

**Significance Statement**

The paper offers a new take on the “fake news” debate. Rather than assuming that election deniers believe that their claims are true, the paper proposes that the statements are made to endow the speaker with a reputation or an identity, like a brand name. This puts the statements in the same category of social signals as men's long hair in the 1960s or women's use of Virginia Slims in the 1970s. The fact that we normally assume that speakers believe in what they say is irrelevant for the signaling function. On the contrary, the obvious falsity means that some others will think poorly of the speakers and this very fact proves that they are serious about the signal sent.

## Introduction

In the 1960s a lot of men grew their hair long, in the 1970s some women smoked Virginia Slims cigarettes, and today many Americans voice the view that the 2020 presidential election was stolen. Each of these actions had three functions: they declare allegiance to one side of a social controversy (a relaxation of social norms, gender equality, and Donald Trump's populism, respectively) and give the actor membership in the “club” (ingroup) of the likeminded, while at the same time causing others to look at them with disdain.^a^ Specifically, many other members of society saw the senders in our three examples as uncivilized, confrontational, and naive, respectively. The willingness with which senders bear these costs proves that they place a high value on expressing allegiance to the cause and gaining membership in the club of those who support it.

The signals are arbitrary in the sense that there is no logical connection between the behaviors and the causes with which they became associated. Many other signals could have worked just as well. However, we can at least conjecture how the connection emerged in each of our three examples. In the Virginia Slims case, Phillip Morris simply paid for ads in which it explicitly suggested the signal conveyed by the long thin cigarettes and its meaning. The company even put an ad in *Playboy* magazine with the text: “We made Virginia Slims especially for women because they are biologically superior to men.” Since this and other ads were seen by a large number of people, it was natural that the suggested equilibrium emerged. Election denial was proposed by a charismatic president, who was able to give it a lot of publicity. The long hair signal took longer to crystalize. It started out among a relatively isolated and unknown subgroup of NYC beatniks but got widespread and positive exposure once it was picked up by popular rock musicians—a very attractive “club.”

On the other hand, not every behavior can serve as a signal. It has to be visible to others on both sides of the social controversy and conflict with widely held norms. In all the three of our lead examples, the behaviors themselves violated existing norms and therefore exposed senders to some social punishments. Players only will engage in the signaling behavior if their utility from publicly supporting the cause plus the value they expect from membership in the club of supporters exceeds the cost of social punishment for deviant behavior. However, they only gain the second benefit if they bear sufficiently high costs. This means that a new sender's willingness to endure those punishments can convince the club of supporters that the newcomer has strong feelings about the cause and club membership, and therefore should be welcomed into their ranks. The costs cannot be too low, which is why the signal—unlike making a true statement—has to be controversial. On the other hand, they also cannot be so high that no one will find the tradeoff worthwhile.

The benefits from membership in the club of supporters depend on the composition of that group. A signal is much more likely to take off if the initial senders are socially attractive and highly visible. However, if the club becomes too popular, people with weaker feelings will want to join and the association between the signaling behavior and support for the cause disappears. For example, by the 1970s long hair was simply fashionable and worn by most men, including many with conservative beliefs.^b^ Both the value of club membership and the costs of punishment also depend on the number of members in the club. A bigger club is more attractive and leaves fewer opponents to punish. The groups administering the social rewards and punishments are not the same for all would be signalers. It depends on who they care about. For example, politicians care about the opinions of voters in their district and many young men worry primarily about friends at school, parents, teachers, and maybe a few others in the local community.

We formalize these arguments in a model in which players decide whether to send a signal by weighing payoffs from three sources: (i) The intrinsic value they place on expressing allegiance to the cause associated with the signal, (ii) the value of membership in the club of supporters, and (iii) the cost of disdain from opponents. Note that (ii) depends on the social attractiveness of pioneering senders (the signal shows that you have the “right” opinions to qualify for membership in the group), while (iii) has to do with the form of the signal (as in “long hair is disgusting”).^c^

In sum, the theory differs from more traditional analyses of social movements because the intrinsic value players place on expressing support for the cause is complemented by their desire to gain membership in an attractive peer group and the social punishments that proves the strength of their feelings. The added forces constitute a theory of self-branding and the predicted behavior emerges as a separating equilibrium of a game. A surprising implication of the model is that the membership benefits for many senders may matter much more than the intrinsic value of expressing support for the cause—they are using borrowed feathers to brand themselves. Another unexpected result is that the signal has to be socially unattractive though neither too much nor too little.

In the rest of the paper, we first discuss related literature with special focus on the fake news issue. After presenting the model in the Model section, we apply it to fake news in the Application to election denial section, propose some testable predictions in the Testable predictions section, and end with a brief summary in the Conclusion section.

## Literature

The present paper uses elements from the theory of branding to develop the view that signals sent in the context of specific social controversies can be seen as players’ attempts to “self-brand” and need no connection to the controversy itself beyond the fact that others use them as such. The two basic building blocks are Benabou and Tirole (1) in economics, and Wernerfelt (2) in branding. Like the former, our model is based on the premise that players value and are willing to incur costs to get peer approval. The key difference is that we are concerned with situations in which two groups hold opposing viewpoints. This means that actions will have both positive and negative reputation effects and that these will interact in a signaling equilibrium. The latter paper is about situations in which brand choice is a signal and looks at equilibria in advertising markets when firms compete to take advantage of the effect exploited by Virginia Slims.^d^ That analysis does not rely on the existence of an underlying social controversy or reputation effects.

While the model's ability to explain “fake news” may have the broadest interest, the width of possible applications means that the paper relates to multiple literatures and thus that any review unavoidably is incomplete. Even though parallels with other signals help sharpen intuition about the mechanics of the model we will, in the interest of brevity, concentrate on the application to fake news.

A lot of literature about fake news has been focused on explaining why people come to have incorrect beliefs. In an early and influential piece, Sunstein (4) observed that people selectively expose themselves to claims that support their initial beliefs, while Pennycook and Rand (5) find that re-senders often fail to take the time to apply appropriate skepticism.

Some recent work has taken a more instrumental view of beliefs. For example, Wagner-Egger et al. (6) suggest that shared beliefs in conspirative, and often false, theories can help unify a group of people all of whom are outside the mainstream. Another line is due to Funkhauser (7) who proposes that (i) people can detect each other's beliefs and (ii) we sometimes take advantage of this by unconsciously changing our beliefs to deceive others—not because we observe new facts. Based on this, Williams (8) uses game theoretic reasoning to argue that players may embrace views that are seen as absurd by outsiders to signal ingroup commitment by willingness to absorb punishment from the outgroup.^e^ Consistent with Funkhauser’s theory, and in contrast to the view presented here, Williams assumes that the beliefs themselves are genuine and function as signals.^f^ In a further development of the signaling perspective, Ganapini (10) goes beyond those two assumptions. She looks are statements as signals and suggests that their primary objective sometimes is to signal membership in a group rather than make claims about the state of the world. Like us, she weighs a negative effect from outgroup punishment against a positive effect from ingroup membership. A key difference between her argument and that made here is that payoff has three components in our model: the personal pleasure derived from expressing support for or otherwise helping the cause, the utility from being accepted as a member of the ingroup, and the social punishment from the outgroup. Our equilibrium may exist without the first, but the model makes the richest set of predictions if all three effects are taken into account. A second difference is that she suggests that some people are particularly inclined to share false, as opposed to true, claims that are favorable to their cause, while we explicitly do not address a choice between true and false statements (since the former cannot have any signaling function). A final difference is that we conduct an explicit game theoretic analysis and therefore can identify the conditions on parameters under which the signaling equilibrium does and does not exist.^g^

One thing missing from our model is any explanation of the social controversy which underlies the difference between the in- and out-groups. It is simply assumed to exist. One possible explanation, suggested by a referee, is based on an implication of the “Green Beard” effect (“I have a green beard and I will be altruistic toward anyone else with a green beard”).^h^ This effect suggests that individuals may be genetically programmed to cooperate in groups that compete against other groups, thus supplying an explanation for the constant supply of social conflicts.

## Model

There is a controversial social cause and a unit mass of players whose opinions fall in *n* different classes with a mass *M_t_* of types *t* ɛ {1, 2, …, *n*}. To keep things simple, we assume that the distribution of masses over types is common knowledge but that the types of individual players are their private information. Not all players understand how to express their support for the cause, but players of type *t* who do so get intrinsic utilities *α_t_* from doing it and we label them opponents if *α_t_ <* 0 and supporters if *α_t_* > 0, while players of type *o* are indifferent and have *α_o_* = 0. Players who do not express their support get 0. That is, the type which is neither an opponent nor a supporter is denoted by the letter *o* which is somewhere between 1 and *n*. The types are labeled such that players of type *t* + 1 are more supportive of the cause than players of type *t.* So the *M*_1_ players of type 1 are the most strongly opposed and the *M_n_* players of type *n* are the most strongly supportive and *α_t_*_+__1_*>α_t_*.

A small group of pioneers have proposed that players can express their support by sending a specific signal. The pioneers are characterized by two different properties: First, their visibility *v* ɛ [0, 1], which equals the probability that a random player is aware that they can express their support for the cause by sending the signal. This means that signals suggested by pioneers with low visibility (low *v*) will be adopted by fewer players. Second, their social attractiveness, which is reflected in the utility experienced by players who send the signal if doing so gives them membership to a “club” with the pioneers and other signalers. Club members socialize with and support each other. Players value membership differently but all value it more if the total number of senders is larger. Specifically, if *X* players signal, those of type *t* value the association with the pioneers as *θ_t_β*(*X*), where *β*: [0, 1] → *R*_+_, *β*′(*X*) > 0 and *θ_t_* ≥ 0.^i^ So *β*( ) is a function describing how the generic value of club membership grows as more players signal, while the *θ_t_* are scalars describing how highly different types value the membership. To keep the formulas uncluttered we here assume that the *θ_t_* are ordered as the *α_t_* such that *θ_t_*_+__1_ > *θ_t_* ≥ 0. This is, however, not necessary.

The signal is characterized by the extent to which it conflicts with existing norms. This is reflected in the disutility experienced by signalers because opponents think poorly of them. It is more costly if there are more of the latter. So if *X* players signal, each bears the cost *γ*(1 − *X*), where *γ*: [0, 1] → *R*_+_, *γ*′(1 − *X*) > 0, such that *γ*( ) is a function describing how nonsignalers’ punishment shrinks as more players signal. Signaling results in membership if club members can infer that the sender feels sufficiently strongly about the cause. The exact meaning of “sufficiently” will vary from case to case, but it will always be defined in terms of a critical type such that acceptance is granted in separating equilibria in which all senders are of the critical type or higher. Since the equilibrium only exists if signalers are granted membership, we will illustrate the process with an example. In particular, we will assume that others want to be sure that any signaler is more likely to be a supporter than an opponent.^j^ With this criterion, the lowest possible critical type, call *h*, satisfies $\sum_{t=h}^{o-1}M_{t}<\sum_{t=o+1}^{n}M_{t}<\sum_{t=h-1}^{o-1}M_{t}$. That is, the mass of players with *α_t_ > 0* is larger than the mass between *h* and *o* − 1 (with *α_t_* < 0) but smaller than the mass between *h* − 1 and *o* − 1. So any separating equilibrium in which all aware players with types *k* ≥ *h* and above send the signal will allow them to make the desired inference. There can be no separating equilibrium if the signal is sent by the aware fraction of players of all types between *k* and *n* for *k* < *h*, since a signaler then is more likely to be an opponent than a supporter.^k^

An equilibrium in which all types higher than or equal to *k* signals while no lower types do so has to satisfy the following three conditions:


(1) $$\alpha_{k}+\theta_{k}\beta \left(v\left[\sum_{t=k}^{n}M_{t}\right]\right)-\gamma \left(1-v\left[\sum_{t=k}^{n}M_{t}\right]\right)>0,$$

(2) $$\alpha_{k-1}+\theta_{k-1}\beta \left(v\left[\sum_{t=k}^{n}M_{t}\right]\right)-\gamma \left(1-v\left[\sum_{t=k}^{n}M_{t}\right]\right)<0,\;\mathrm{and}$$

(3) kϵ{h,h+1,…,n}.

In words, in a separating equilibrium in which all types ≥ *k* signal and no types lower than *k* do so, it has to be true that, if all other players follow their equilibrium strategies, (Eq. 1) type *k* gets positive net payoffs from signaling whereas (Eq. 2) type *k* − 1 does not. Finally, (Eq. 3) type *k* cannot be so low that more opponents than supporters send the signal.

Since *α_t_* and *θ_t_* are increasing in *t*, if type *k* − 1 does not signal neither does any lower type. Similarly, if type *k* does signal, so do all higher types. The equilibrium therefore exists if Eqs. 1–3 hold.

We can combine Eqs. 1 and 2 to identify an important property of the equilibrium.


(4) $$\alpha_{k}+\theta_{k}\beta \left(v\left[\sum_{t=k}^{n}M_{t}\right]\right)>\gamma \left(1-v\left[\sum_{t=k}^{n}M_{t}\right]\right)>\alpha_{k-1}\;+\theta_{k-1}\beta \left(v\left[\sum_{t=k}^{n}M_{t}\right]\right).$$

(Eq. 4) shows that the punishment inflicted by nonsignalers can neither be too small nor too large and that the same is true for the positive incentives to signal and the visibility of the pioneers.

**Fig. 1.**
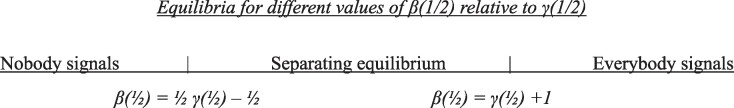
Equilibria for different values of *β*(1/2) relative to *γ*(1/2).

To see how this works in an example, suppose that *n* = 2, *M*_1_ = *M*_2_ = 1/2, *α*_1_ = − 1, *α*_2_ = 1, *ν* = 1, *θ*_1_ = 1, *θ*_2_ = 2, and *β*(1/2) = *γ*(1/2), such that the functions *β*( ) and *γ*( ) have the same value at 1/2. In this example, Eq. 1 is 1 + *β*(1/2) > 0, while Eq. 2 is −1 < 0. So we have an equilibrium in which type 2 signals, while type 1 does not. However, if the ingroup is very attractive such that *β*(1/2) = *γ*(1/2) + 2, Eq. 2 becomes 1 > 0 and type 1 will break the equilibrium and signal as well. There is also no separating equilibrium if the outgroup punishment is very severe such that *γ*(1/2) = 2*β*(1/2) + 2, since Eq. 1 then becomes −1 < 0 and no one wants to signal. Figure 1 illustrates this in the context of the example.

It is possible that there is more than one separating equilibrium. There could, for example, be an additional equilibrium at *k* + 1, because Eq. 1 may hold simultaneously with


(5) $$\alpha_{k+1}+\theta_{k+1}\beta \left(v\left[\sum_{t=k+1}^{n}M_{t}\right]\right)-\gamma \left(1-v\left[\sum_{t=k+1}^{n}M_{t}\right]\right)>0,\;\mathrm{and}$$

(6) $$\alpha_{k}+\theta_{k}\beta \left(v\left[\sum_{t=k+1}^{n}M_{t}\right]\right)-\gamma \left(1-v\left[\sum_{t=k+1}^{n}M_{t}\right]\right)<0.$$

Summarizing,

### Proposition

If Eqs. 1–3 hold, there exists at least one separating equilibrium in which all aware players of types *k* or higher send the signal and no other players do.

What does Eqs. 1 and 2 tell us?

- Larger or smaller values of *v* cause more or fewer players to signal and may even lead to violation of Eq. 3 such that the signal loses it meaning or never takes off. These scenarios occur when the pioneers have very high or low visibility, respectively.
- Shifts in *θ* and *β*( ) have much the same effect. If the “club” of signalers is more or less attractive, larger or smaller numbers of players will want to join it. So the signal has meaning and is used for intermediate values of *θ* and *β*().
- The equilibrium also does not exist if *γ*() is too small or too large. Players earn the right to join the club because their willingness to endure *γ*() proves that their types, measured by *α_t_* and *θ_t_* are high. However, *γ*() cannot be so large that no one wants to take it on. Conversely, a downward shift in *γ*(), for example, because the cause gains social support, causes more players to signal.
- When more types signal, the average sender feels less strongly about the cause.

Our three leading examples can be interpreted in this light. The long hair for young men went mainstream (the separating equilibrium ceased to exist) because men opposed to the cause realized that a lot of young women liked the rock star look such that the net reputational effect became positive. Conversely, Virginia Slims lost sales and market share because Phillip Morris cut back on advertising spending as the social punishment for smoking grew.^l^ There are no feminist messages in today's ads and the signal would now seem to at best be very weak. In contrast, election denial remains in a separating equilibrium as of 2024: It has become *the way* to identify yourself as a MAGA supporter; no one opposed to the cause would say it but almost all supporters will. We will now look at this in greater detail.

## Application to election denial

A key implication of the theory is that election denial should not be seen as an attempt to argue for the veracity of the claim but simply as a way to express the speaker's allegiance to the broader MAGA agenda. ^m^ It can only function as a signal because it is false but that does not mean that false statements are preferred. It is possible, and in light of Ghezae et al. (11) likely, that people prefer making true statements. However, that does not mean that they will make no false ones.

The calculus behind their use of the election denial signal is slightly different for republican politicians and regular voters who resend the signal. The former group wants to avoid the negative electoral consequences of not signaling. The politicians’ hoped-for reward for signaling seems to be that they will be accepted in the “Trump club” and elected. The punishment is that others may see them as “sell-outs” (in the signaling interpretation) or as naïve (in the literal interpretation).

For nonpoliticians the reputational effects of social media activity and in-person statements are mostly confined to their personal network. Beyond supporting the MAGA agenda, the reward could be acceptance into a local and online groups of staunch republicans with accompanying friendships and socialization (see also Footnote a). The punishment would be that some others in their personal network consider them naïve victims of propaganda or even stupid. In contrast to the politicians, it may be possible for individual voters to hide their statements from some or all of those likely to punish. However, not all of them would want or be able to do that. Some might value spreading the MAGA agenda, some might fear that they would lose the reputational benefit if they were accused of selective communication, some may find it too cumbersome to send direct messages as opposed to simply posting, and no one can fully control the further actions of those to whom they send or in the presence of whom they speak. If some, but not all, signalers manage to avoid much punishment, the model still applies to all senders as long as the ingroup cannot tell who does and does not avoid punishment. It just means that the credibility condition has to take this into account. Specifically, suppose that a fraction *p* of the population can signal and escape punishment by sending the signal to the ingroup and, without the knowledge of that group, not sending it to the outgroup. A type *t* player in this fraction will signal if *α_t_* + *θ_t_β*(*X*) > 0, so if we label the smallest type for which this is true as *s*, then this group will include *p*$\sum_{t=s}^{o-1}\;M$*_t_* opponents and *p*$\sum_{t=o+1}^{n}\;M$*_t_* supporters. Going on to the fraction of players who cannot escape punishment and looking for a separating equilibrium, we continue to use *k* to denote the lowest type to signal. In that case, there will be (1 − *p*) $\sum_{t=k}^{o-1}\;M$*_t_* opponents and (1 − *p*) $\sum_{t=o+1}^{n}\;M$*_t_* supporters among the signalers in the latter group. Again assuming that the ingroup will accept a signaler if more than half of all signals are sent by supporters, *k* has to satisfy either *p*$\sum_{t=s}^{o-1}\;M$_t_+$(1-p)\sum_{t=k}^{o-1}\;M$*_t_* < $\sum_{t=o+1}^{n}\;M$*_t_* or *p*$\sum_{t=s}^{o-1}\;M$*_t_ < p*$\sum_{t=o+1}^{n}\;M$*_t_* +(1 − *p*) $\sum_{t=k}^{n}\;M$*_t_*. So an equilibrium can exist if *p*$\sum_{t=s}^{o-1}\;M$*_t_* < $\sum_{t=o+1}^{n}\;M$*_t_*. The model is consistent with players in both fractions signaling but it is the second fraction for which the explanation is most interesting.

## Testable predictions

One way to test the model is to use (i), (ii), and (iii) from “Model” to look at the birth and death of signals over time. We do not observe signals that never take off, but we sometimes have signals, such as the long hair, which are proposed long before they ever take off. For such signals the takeoff should follow upward shifts in *v*, *α, θ*, and *β*(), downward shifts in *γ*(), or a combination of those. Similarly, signals that lose their meaning because they become ubiquitous, also like the long hair, should do so after one or more of the same shifts. On the opposite side, signals that die off because no one wants to use them anymore, should do so after downward shifts in *ν*, *α*, *θ*, and *β*(), upward shifts in *γ*(), or a combination thereof. We could compare the pre- and post-shift equilibria and test (iv). Finally, if the same signal is used in multiple isolated populations, we may be able to compare across equilibria.

If we consider a simple dynamic version of the model with myopic players, it could also be possible to test the logic by comparing adopters over time. Depending on the specifications, many natural versions of such a model with myopic adopters will predict that *v* and *β*() grow over time while *γ*() declines. This again means that higher types, who feel more strongly about the cause, should adopt sooner than lower types and that the average type falls over time.

## Conclusion

We have proposed a model in which players take symbolic actions in part to announce their stand on a social controversy but also, and maybe mostly, to gain reputational benefits by joining socially attractive groups. The “fake news” debate is an important application of the model: Rather than asking whether the proponents believe that their claims are true, it suggests that they may be engaging in self-branding for social gain. In fact, the model's predictions do not depend on the beliefs of the proponents.

## Supplementary Material

pgae408_Supplementary_Data

## Data Availability

No data was used.

## Notes

a It is not hard to find examples in which a person’s opinions influence how others treat them: Many Americans would not socialize with individuals who support particular aspects of the opposite political ideology (e. g. about global warming, abortion, or gun rights). Similarly with religion, morality, race relations, etc. Conversely, people are often eager to talk with strangers if they believe them to be like-minded about those same topics.

b Other examples of uninformative signals include the hand gesture used in Nazi Germany and proclamations of support for dictators.

c This paper was born when the author overheard a young man talking about the social advantages of voicing the “right opinions.”

d This literature also has work on how brands come to be endowed with consumer-like personalities (e.g. Huang et al. (3)).

e This is also consistent with Zahavi’s (9) “Handicap Principle.”

f The parallel with other signals drives this point home: it does not make sense to talk about beliefs in long hair or a brand of cigarettes.

g Ghezae et al. (11) test Ganapini’s argument and find that, contrary to her predictions, the increase in utility from sharing a true versus a false claim is the same for claims that are favorable for the cause and claims that are unfavorable for the cause. So they find no support for the idea that partisans are particularly inclined to share false stories that are favorable to their cause. These findings are not inconsistent with our model since a separating equilibrium does not exist for true statements. (So we simply do not address peoples’ motivations for making them.) The question we ask is whether a false statement, if available, will be shared. We do not look at a choice between true and false statements. This does, perhaps, illustrate the value of formal models.

h The underlying “Selfish Gene” theory was originally proposed by Dawkins (12) but considered unproven until Keller and Ross (13) found an example.

i It is worth noting that our results hold if the *α_t_* are identically 0. However, in the general case, they will not be.

j This is just an example; it could also be that say 80% of all signalers are supporters.

k A list of symbols is given in Supplementary material.

l I am indebted to an anonymous reviewer for this interpretation.

m The model does not depend on the beliefs of the senders, but it provides an explanation for people knowingly making false statements. In the case of the politicians, we know from their earlier statements that many of them at least initially believed that the election was not stolen. So, it is hard to see their current claims as representing beliefs about the state of the world.
