# Supplementary material for: The origin and function of arbitrary signals: Making false statements, having long hair, and smoking Virginia Slims
Source: PNAS Nexus. 2024 Sep 14;3(9):pgae408. doi: 10.1093/pnasnexus/pgae408 (PMC11428180; doi:10.1093/pnasnexus/pgae408)
Supplement: pgae408_Supplementary_Data [file pgae408_supplementary_data.docx]

**SUPPLEMENTARY MATERIAL**

*List of symbols*

*t is a type of player.*

*n is the number of types.*

*M_t_ is the mass of players of type t.*

*α_t_ is type t’s intrinsic utility from signaling.*

*o is the type for which α_t_ = 0 (so α_o_ = 0).*

*ν is the visibility of pioneers.*

*β(X) is the generic value of club membership if X players signal.*

*θ_t_ β(X) is the value of club membership for type t.*

*γ(1 – X) is the cost of punishment from non-signalers if X players signal.*

*k is the lowest type to signal in a generic separating equilibrium.*

*h is the lowest possible value of k.*
